# Traumatic neuroma of the superficial peroneal nerve in a patient: a case report and review of the literature

**DOI:** 10.1186/s12957-016-0990-6

**Published:** 2016-09-10

**Authors:** Jian Kang, Pinglin Yang, Quanjin Zang, Xijing He

**Affiliations:** Second Department of Orthopedics, Second Affiliated Hospital of Xi’an Jiaotong University Medical School, Xi’an, Shaanxi Province China

**Keywords:** Traumatic neuroma, Superficial peroneal nerve, Neuralgia

## Abstract

**Background:**

Traumatic neuromas are rare benign tumors, which are common in trauma or post-operation and accompanied with obvious symptoms of pain. This study will show the superficial peroneal nerve neuroma occurring after resection of hemangioma.

**Case presentation:**

A 44-year-old male had an operation of the right leg cavernous hemangioma resection in 1995. Half a year after the operation, pain around the wound appeared and gradually aggravated. The patient had the lesion exploration resection in 2013, and the pathological result showed traumatic neuroma. Within half a year of the second operation, severe pain showed up again, so neuroma resection proceeded in May 2015. The postoperative pathological and immunohistochemical results showed traumatic neuroma. According to the postoperative follow-up, there were no symptoms of pain appearing again.

**Literature review:**

The pain is obvious, and B ultrasonography is the most efficient way to find neuromas. Both conservative and operative therapy have their advantages and disadvantages.

**Conclusions:**

There remain many unanswered questions in relation to the treatment of traumatic neuromas, and further research is required, although we have already had adequate understanding of traumatic neuromas.

## Background

Traumatic neuromas are rarely seen in clinical practice but are common in trauma or post-operation. The main clinical manifestations are pain and paresthesia. Most scholars consider the occurrence of traumatic neuroma to be related to excess hyperplasia and irregular hyperplasia after nerve injury, so they are often regarded as benign tumors [1]. Most reports show neuromas in the face, neck, and limbs, and the superficial peroneal nerve neuroma has not yet been reported in English literature at present. The purpose of this article is to show the details of a case of superficial peroneal neuroma after the operation of hemangioma, including clinical manifestation, clinical examination, diagnosis, and treatment, and review the relevant literature and which remain unanswered in the current literature.

## Case presentation

**Fig. 1:**
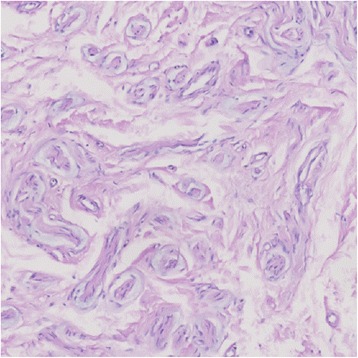
The pathological image of the lesion in the second operation

A male, 44 years old, had an operation of hemangioma resection, because of the right leg cavernous hemangioma in 1995. Six months later, the patient reported increased pain in the original surgical site, radiating to both the upper and lower sides, and further aggravated with the rising of temperature. In 2013, the hemangioma recurrence was taken into account, so lesion exploration resection was performed. The pathological result showed traumatic neuroma (Fig. 1). Six months after the second operation, the pain appeared again, which the patient reported as persistent, radiating into the inner thigh, groin, and foot. The pain significantly increased upon touching and percussion, accompanying with numbness. The patient could still walk, but with intermittent claudication. The conservative treatment such as antidepressant, antispasmodic, NSAIDs, and neurotrophic drugs had been given with no obvious effects.

**Fig. 2:**
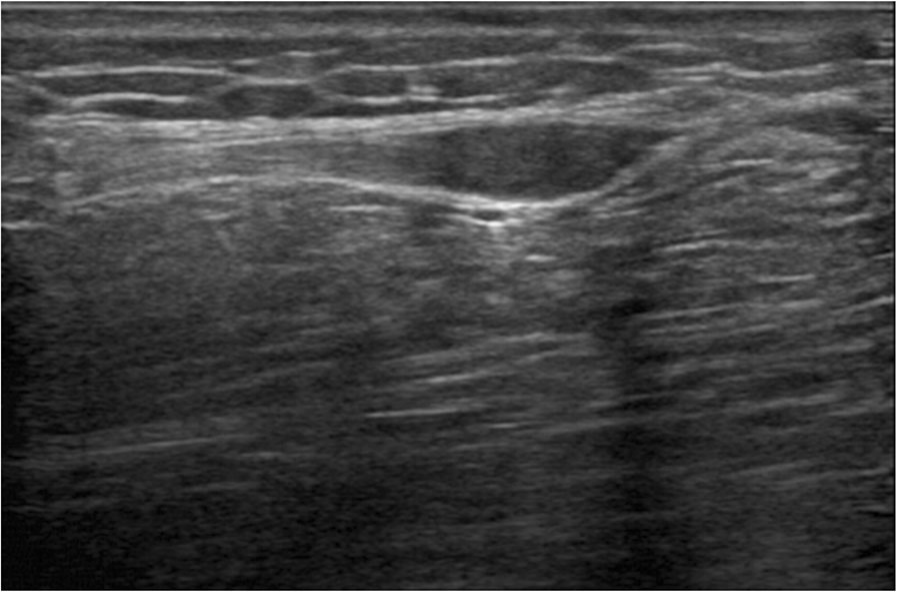
B ultrasound: a 12.0 × 5.0 mm fusiform hypoechoic nodule can be found between the right calf fat depth layer and muscle layer, with clear boundary and both ends connected to the fiber ribbon cord-like structures

**Fig. 3:**
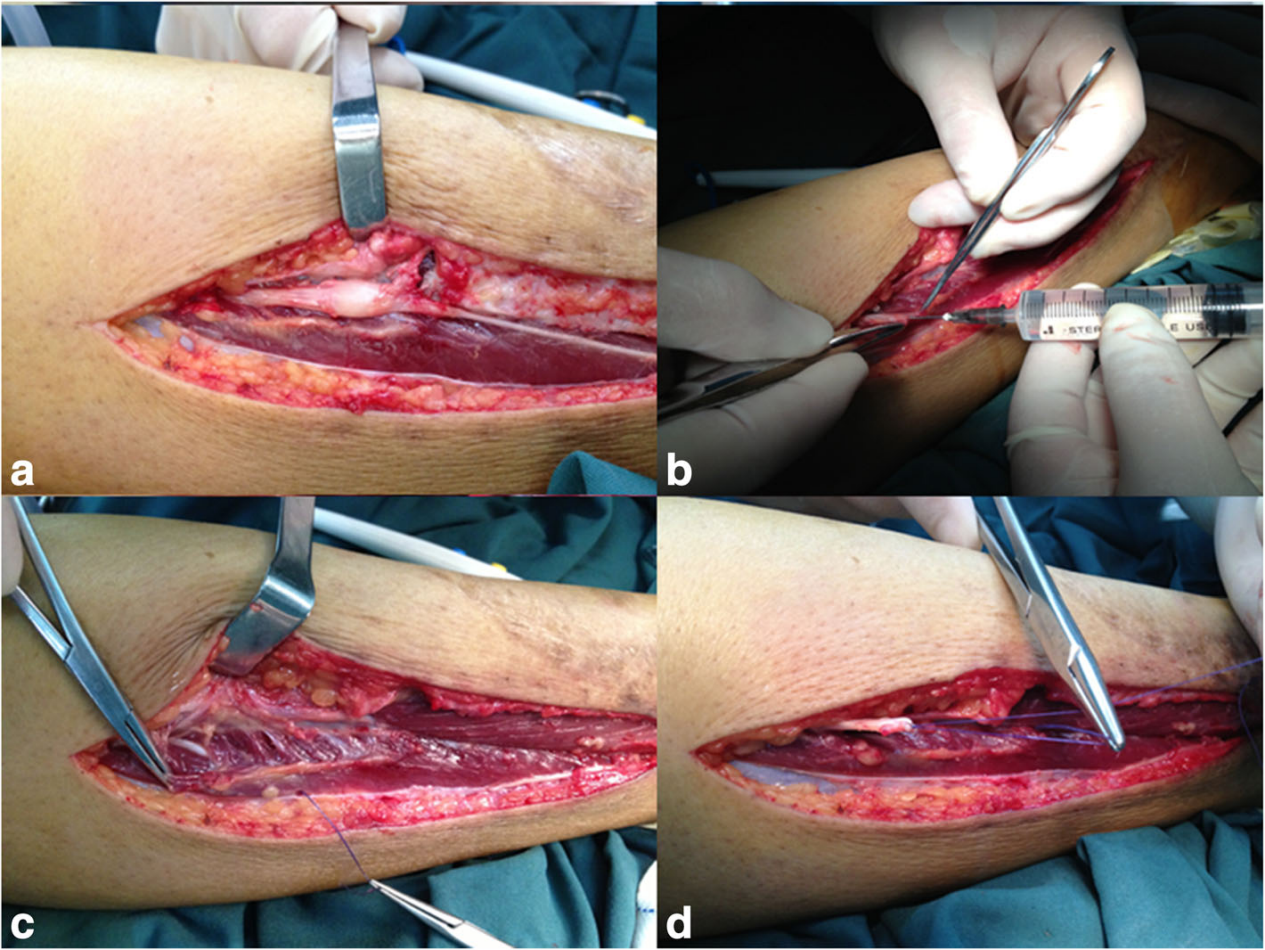
During the operation, **a** two fusiform masses were founded in superficial peroneal nerve trunk, with smooth surface, and with the inner side and front side closely connected to the subcutaneous tissue and deep fascia and unable to be completely separated. **b** Injection of 1 % lidocaine to the stump after completely resected the neuroma. **c** The stump was embedded in the long peroneal muscle. **d** Suturing and wrapping the stump by the epineurium

B ultrasound: a 12.0 × 5.0-mm fusiform hypoechoic nodule can be found between the right calf fat depth layer and muscle layer, with clear boundary and both ends connected to the fiber ribbon cord-like structures (may be from the superficial peroneal nerve) (Fig. 2). Hence, the neuroma excisional biopsy was performed, and two fusiform masses about 12.0 × 5.0 mm and 10.0 × 2.0 mm were founded in superficial peroneal nerve trunk during the operation (Fig. 3a). Completely resected the neuroma, we then injected 1 % lidocaine to the stump (Fig. 3b), which was embedded in the long peroneal muscle (Fig. 3c) after suturing and wrapping the stump by the epineurium (Fig. 3d). Finally, the local scar tissue was removed. The patient was followed up for 2 months after the operation, and the pain completely disappeared.

### Specimen

**Fig. 4:**
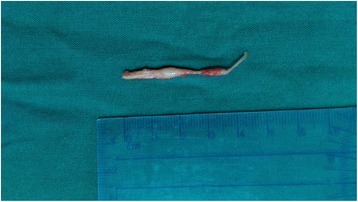
Gross appearance of traumatic neuroma: two separately fusiform masses about 12.0 × 5.0 mm and 10.0 × 2.0 mm are connected to the upper and lower nerves

**Fig. 5:**
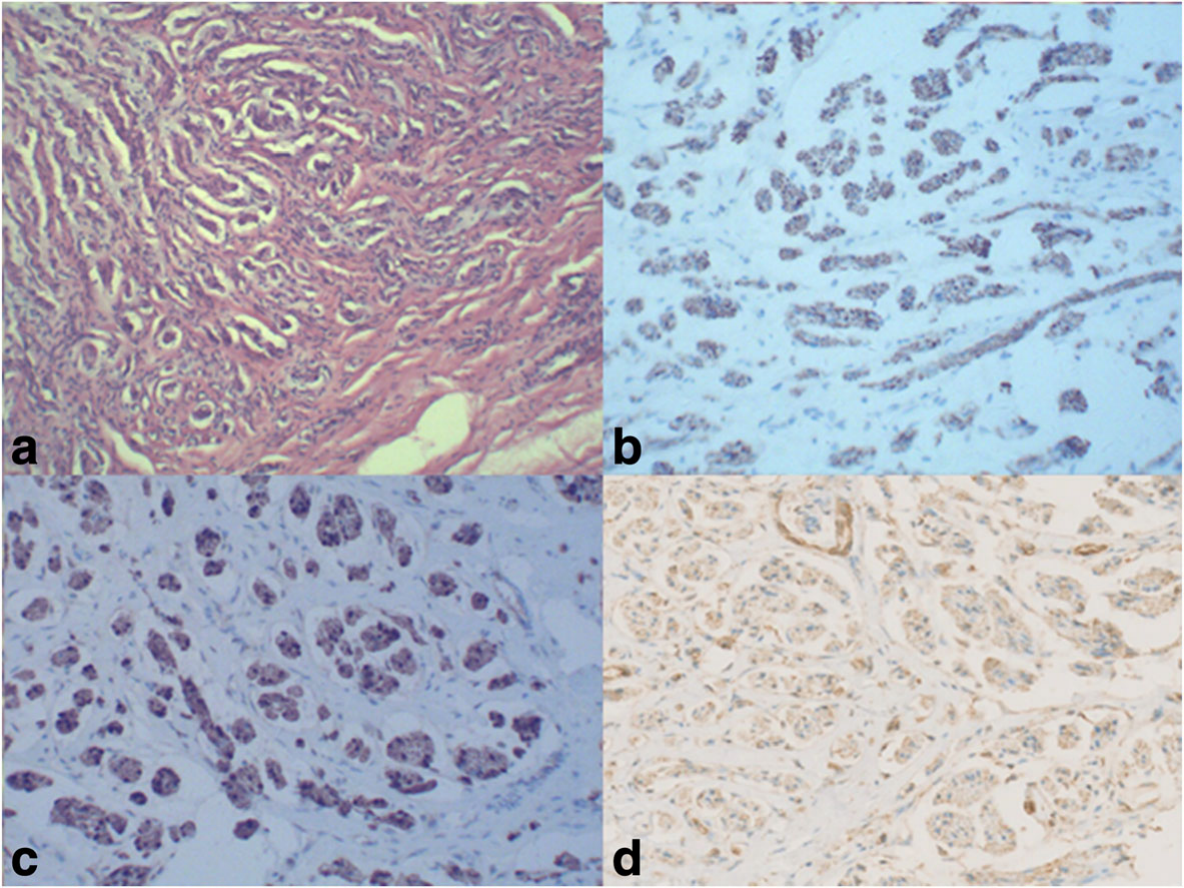
Pathology and immunohistochemistry images of traumatic neuroma: **a** hematoxylin and eosin (HE) stain shows a haphazard, tortuous arrangement of nerve bundles within a fibrous connective tissue stroma; **b**–**d** S100, NF, and VIM highlight the nerve bundles

Gross appearance: two separately fusiform masses about 12.0 × 5.0 mm and 10.0 × 2.0 mm are connected to the upper and lower nerves, with smooth surface, clear boundary, and tough texture, and adhere to peripheral scar tissue (Fig. 4). Pathology: traumatic neuroma (Fig. 5a). Immunohistochemistry: S100, NF, Vim, EMA lesion, masson, VG (+) (Fig. 5b–d).

## Review

Traumatic neuroma is very rare and seldom reported in English literature. Relatively, many traumatic neuromas occurring in the limbs, face, and neck can be found in the current literatures, and studies have also reported that traumatic neuroma could occur in the mammary gland, penis, and other body parts [1–4]. As discovered, most of the cases occurred secondary to direct trauma or operation. Currently, most scholars believe that this kind of lesion results from excessive repair and excess hyperplasia of oneself after nerve injury, so they do not consider it as tumor in situ [5–7]. Traumatic neuroma can be roughly classified into two categories. One is called terminal neuromas, which commonly occur in injured or divided proximal nerve terminal after injury or operation. After nerve division, distal axons begin apoptosis. To reconstruct nerve continuity, Schwann cells of the distal nerve will generate a channel for ingrowth of adjacent axons [8]. However, in cases of too long distance between two segments of the injured nerve, proximal axons will grow toward multiple directions around so as to find a way for growth; thus, the appearance generally overgrows in bulbous-end-like shape [8], and besides, as nerve tissues grow more slowly than surrounding soft tissues, they will be mixed with other kinds of cells, such as fibroblast and mastocyte [9]. The other one is spindle neuromas, which commonly occur in complete nerves and currently are considered as resulting from chronic stimulation and friction [10]. There is also a report indicating that facial nerve hemangioma also can lead to traumatic neuroma under the condition of no trauma or operation history and no chronic stimulation and compression [4].

The generating mechanism of superficial peroneal neuroma reported in this study may be the reactive hyperplasia of the nerve. The injury is exactly at the branch of superficial peroneal nerve, reactive hyperplasia occurs in proximal end and further extends toward the trunk, thus generating eccentric neuroma occurring in superficial peroneal nerve trunk. In addition, we consider that the compression induced by scarring of surrounding soft tissues is also an important cause of traumatic neuroma.

### Clinical manifestation

The main manifestation of traumatic neuroma is pain, especially intense neuralgia [8, 11]. The pain may be generated for the following reasons or mechanisms: (1) stretched by surrounding scar tissues [12]; (2) compression on sensitive teleneuron by surrounding soft tissues [13]; (3) nerve tissue ischemia [14]; (4) ectopic foci of ion channels [15, 16]; (5) P substance, calcitonin gene related peptide and 5-hydroxytryptamine are released by mastocyte [9, 17]; and (6) peripheral regulation of kinds of ion channels or receptors (sodium channels, TRPA1, alpha1C receptors, and nerve growth factor) [15, 18–21]. The pain is often characterized by chixuxi low-intensity dull pain or intense paroxysmal burning pain and also can be induced by various outside simulations, such as temperature and touch. Tinel’s sign is usually positive [22]. This kind of pain is difficult to control because it is often accompanied by central sensitization and social-psychological factors [23], and the change of voltage-gated calcium channels (VGCCs) may be the important element of central sensitization [24]. There is also another pain that may be related to traumatic neuroma, called phantom pain, which results from sensing of the existence of amputated part [25]. Ectopic discharge from a stump neuroma has been thought to be an important peripheral mechanism of phantom pain [25]. Traumatic neuroma may also be characterized by paresthesia, such as hyperesthesia or feeling of numbness. One study reports on a case traumatic neuroma may have no manifestation during its development [8, 26].

### Auxiliary examination

MRI is only used in differential diagnosis with other soft tissue diseases [27], and its cost is high; patients have discomfort and real-time imaging cannot be used for display; thus, MRI has a limited value in diagnosis of traumatic neuroma.

The position and size of lesion and the relation between lesion and surrounding tissues and nerves can be detected directly via ultrasonography examination. Ultrasonography is one of most valuable methods in the diagnosis of traumatic neuroma. On one hand, ultrasonography examination is inexpensive, noninvasive, and non-harmful. And on the other hand, local nerve blocking can be conducted under the guidance of ultrasonography for the enhancement of the diagnosis, and pathological examination of living tissue can be implemented for the definite diagnoses [8, 28–30]. Ultrasound image of traumatic neuroma is generally oval, low-echo enclosed mass, clear and irregular probable boundary, diameter being greater than that of nerve trunk, and continuity with nerve [8]. At histopathology, traumatic neuromas generally show in the following appearance: nonencapsulated tangled mass formed by Schwann cell, endoneurial cell, perineurial cell, and surrounding fibroblast [10].

### Therapeutic schedule

The treatment of traumatic neuroma is still considered controversial. Conservative and operative therapy both have its advantage and disadvantage, which are listed in following content.

### Conservative therapy

(1) Pharmacotherapy: Opioid analgesics, antidepressant drugs, antispasmodic drugs, α receptor blockers, and lidocaine are all used in the treatment of traumatic neuroma, but only have short-term therapeutic effect, and show significant side effects and declined therapeutic effect in long-term use [31–33]. Gabapentin and pregabalin are considered the effective medicine to inhibit central sensitization through affecting the calcium channels and reduce the excessive neurotransmitter release [34]. (2) Repeated injection of lidocaine-hormone: It shows the symptom is improved in painful neuroma [35] but has significant side effects with long-term injection. (3) B ultrasound-guided percutaneous ethanol injection: can relieve painful neuroma significantly with continuous injection, and has high remission rate [36]. (4) Transcutaneous magnetic stimulation (TMS): a rapid discharge of electric current is converted into dynamic magnetic flux for modulating neuronal functions [37]. This analgesic effect seemed to be sustainable with repeated treatment delivered at a 6- to 8-week duration [37]. (5) Injection of a tumor necrosis factor α blocker-enalapril to peripheral nerves can relieve the pain significantly [38]. (6) Cryotherapy and radiofrequency ablation are both used in clinical treatment, but the therapeutic effect is unsatisfactory. Six months after radiofrequency ablation, there was significant decline in pain relief [39].

### Operative therapy

(1) Neuroma resection: Simple neuroma resection tends to have short-term therapeutic effect, together with relapse of neuroma [40]. (2) Neuroma resection and ethanol injection: Appears to be only effective for patients who only suffer from short pain before operation, one-time nerve injury and one neuroma [26]. (3) Neuroma resection and targeted nerve implantation (TNI): Such surgical method has been performed many times, and it is speculated to have a great role for prevention and treatment of neuroma [41]. Its mechanism is as such: The proximal nerve ending at a secondary motor point is transplanted to an adjacent denervated muscle, and the regenerated axons may bifurcate and enter the myenteric motor nerve branches, so as to prevent formation of traumatic neuroma [41].

## Conclusions

Adequate understanding of the traumatic neuroma appears essential. However, there remain many problems which require future research, such as (1) Should further remedial measures such as B ultrasound-guided percutaneous ethanol injection be taken after traumatic neuroma is confirmed by postoperative pathological report?; (2) Is it (traumatic neuroma) recurrent? Will it occur in sensory branch or motor branch?; (3) Does it develop to proximal trunk which is not adjacent with injured sites?; and (4) Is it useful to inject lidocaine when the nerve stump is closed? Further follow-up for condition of the patient and research is required.
